# miR-346 Controls Release of TNF-α Protein and Stability of Its mRNA in Rheumatoid Arthritis via Tristetraprolin Stabilization

**DOI:** 10.1371/journal.pone.0019827

**Published:** 2011-05-17

**Authors:** Noha Semaan, Laurent Frenzel, Ghada Alsaleh, Guillaume Suffert, Jacques-Eric Gottenberg, Jean Sibilia, Sebastien Pfeffer, Dominique Wachsmann

**Affiliations:** 1 Laboratoire Physiopathologie des Arthrites, EA4438, Université de Strasbourg, UFR Sciences Pharmaceutiques, Illkirch, France and Departement de Rhumatologie, Hôpitaux Universitaires de Strasbourg, Strasbourg Hautepierre, France; 2 Architecture et Réactivité de l'ARN, Université de Strasbourg, Institut de Biologie Moléculaire et Cellulaire du CNRS, Strasbourg, France; Massachusetts General Hospital and Harvard Medical School, United States of America

## Abstract

TNF-α is a major cytokine implicated in rheumatoid arthritis. Its expression is regulated both at the transcriptional and posttranscriptional levels and recent data demonstrated that miRNAs are implicated in TNF-α response in macrophages. LPS-activated FLS isolated from RA patients express TNF-α mRNA but not the mature protein. This prompted us to look for miRNAs which could be implicated in this anti-inflammatory effect. Using a microarray, we found two miRNAs, miR-125b and miR-939 predicted to target the 3′-UTR of TNF-α mRNA, to be up-regulated in RA FLS in response to LPS, but their repression did not restore mature TNF-α expression in FLS. We showed previously that miR-346, which is upregulated in LPS-activated FLS, inhibited Btk expression that stabilized TNF-α mRNA. Blocking miR-346 reestablished TNF-α expression in activated FLS. Interestingly, transfection of miR-346 in LPS-activated THP-1 cells inhibited TNF-α secretion. We also demonstrated that TTP, a RNA binding protein which inhibited TNF-α synthesis, is overexpressed in activated FLS and that inhibition of miR-346 decreases its expression. Conversely, transfection of miR-346 in LPS-activated THP-1 cells increased TTP mRNA expression and inhibited TNF-α release. These results indicate that miR-346 controls TNF-α synthesis by regulating TTP expression.

## Introduction

One of the key players in rheumatoid arthritis (RA) is TNF-α and therapies targeting this cytokine have already proved beneficial [1]. In RA, TNF-α is produced by many cell types, mainly by macrophages and dendritic cells in response to interactions between pathogen-associated molecular patterns (PAMPs) or damage-associated molecular patterns (DAMPs) and pattern-recognition receptors (PRRs) or to the cytokine environment [2].

The resident cells of the joint space, the fibroblast-like synoviocytes (FLS,) play a crucial role in RA [3], [4], [5]. They are implicated in the inflammatory response essentially by synthesizing cytokines, chemokines, prostanoids, nitric oxyde (NO), and pro-angiogenic factors FLS play a key role in osteoarticular destruction and take also part in the differentiation and activation of osteoclasts by the RANK-RANK ligand pathway, and through the release of PGE-2 and IL-6 [6], [7], [8]. However RA FLS secrete no TNF-α, a major cytokine implicated in RA [9], but intriguingly they express TNF-α mRNA in response to LPS. This was also observed with osteoarthritis (OA) and trauma FLS (unpublished data). It was therefore of interest to identify the molecular basis of this anti-inflammatory mechanism. Post-transcriptional regulation of TNF-α expression depends on AU-rich elements (ARE) located in the 3′-untranslated region (UTR) of TNF-α mRNA. RNA binding proteins such as tristetraprolin (TTP), T-cell intracellular antigen (TIA-1) and T-cell intracellular antigen-related protein (TIAR) can bind to ARE of TNF-α mRNA inducing mRNA lability or inhibition of translation [10], [11]. Mice lacking TTP develop a systemic inflammatory syndrome characterized by cachexia, dermatitis, erosive arthritis and myeloid hyperplasia [12]. Besides the direct destabilization of mRNA by regulatory proteins, another mode of regulation involves miRNAs [13]. MiRNAs are an evolutionarily conserved class of endogenous small non-coding RNAs. They are processed from long primary transcripts by the ribonuclease Drosha in association with DGCR8. After being transported into the cytoplasm, the pre-miRNA is further processed by Dicer and its cofactor TRBP. One strand is then assembled in the RISC complex which always contains a member of the Argonaute family. The miRNA then guides the RISC complex to its target 3′-UTR leading to a decrease of mRNA stability or inhibition of translation [14], [15]. Regulation of miRNAs expression is controlled at the level of transcription, processing and subcellular localization and several recent studies have indicated that these processes can be influenced by factors such as inflammation [16], [17]. Since their discovery, miRNAs have been implicated in a wide array of cellular and developmental processes and emerging data have identified an important contribution of miRNAs to the development and control of the inflammatory response [18]–[21]. For example, Tili et al. [22] demonstrated that up-regulation of miR-155 in LPS-activated macrophages resulted in an enhanced translation of TNF-α mRNA. Mice overexpressing miR-155 produce more TNF-α when challenged with LPS. By contrast, they found miR-125 to be implicated in the posttranscriptional repression of TNF-α mRNA, hence the need for its down-regulation for TNF-α production. A negative correlation between miR-146a expression and TNF-α release was also demonstrated in THP-1 cells [23], [24]. These data indicate that miRNAs can exert both negative as well as positive effects in inflammatory pathways and this prompted us to look for miRNAs which could be implicated in the anti-inflammatory effect of LPS in RA FLS. We found two miRNAs, miR-125b and miR-939, which were predicted to target the 3′-UTR of TNF-α mRNA, to be up-regulated in RA FLS in response to LPS, but their repression did not restore either intracellular expression of TNF-α or its release by LPS-activated FLS. We further demonstrated that inhibition of miR-346, a negative regulator of Bruton's tyrosine kinase (Btk) expression, reestablished mature TNF-α expression in LPS-activated FLS by blocking TTP expression. In parallel, over-expression of miR-346 in LPS-activated THP-1 cells increased the expression of TTP which resulted in inhibition of TNF-α synthesis. These results indicate that miR-346 controls TNF-α synthesis by regulating TTP expression and can therefore act as a negative regulator of inflammation.

## Results

### LPS does not induce TNF-α production by RA FLS

**Figure 1:**
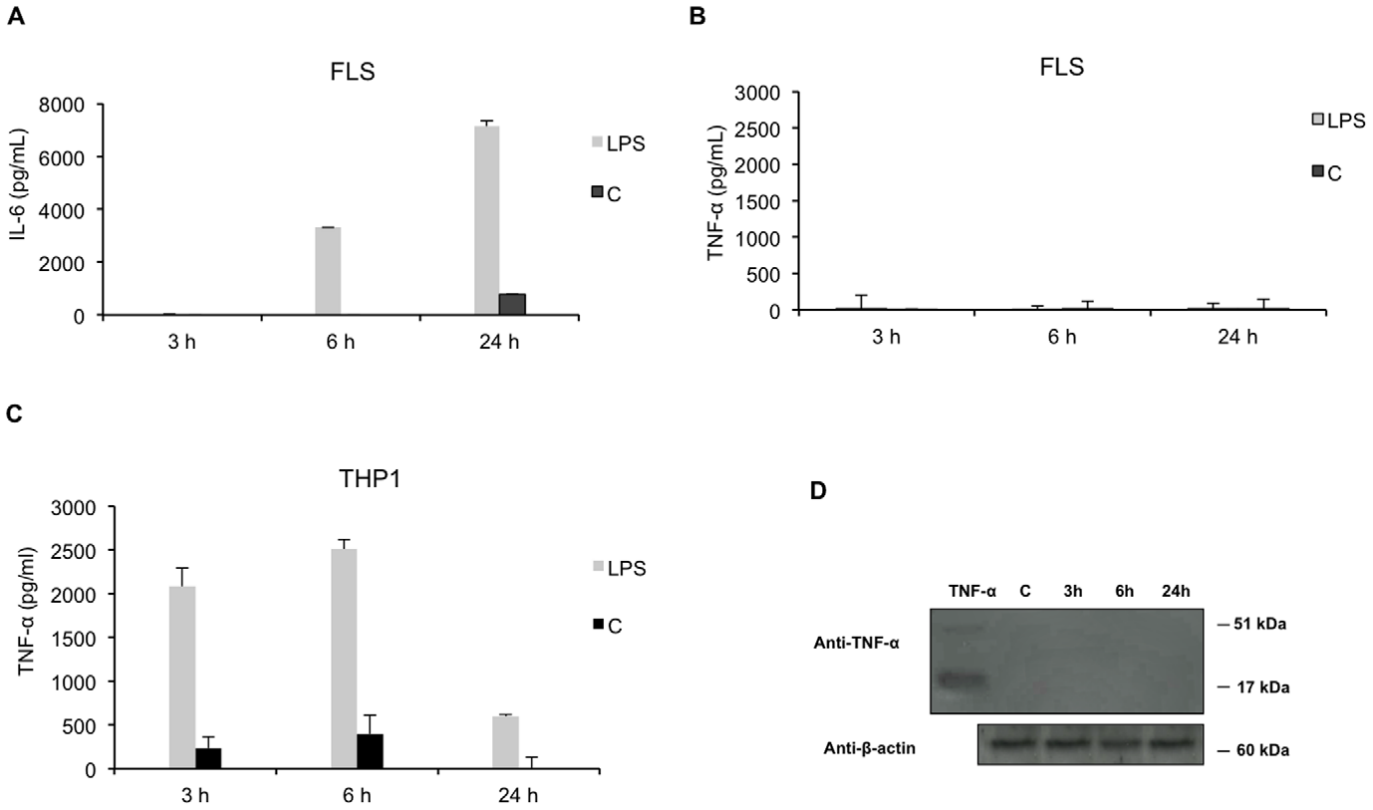
Effect of LPS on TNF-α release by RA FLS and THP-1 cells. **A**. IL-6 release was determined by ELISA in culture supernatants harvested 3 h, 6 h and 24 h after stimulation with LPS (1 µg/ml) or medium (C). **B, C**. TNF-α release by RA FLS and THP-1 cells was determined by ELISA in culture supernatants harvested 3 h, 6 h and 24 h after stimulation with LPS or medium (C). Data are expressed as the mean of triplicate samples ± SD and are representative of three independent experiments. **D**. TNF-α expression was determined by western-blotting with anti-TNF-α antibodies in RA FLS, 3 h, 6 h and 24 h after stimulation with LPS or medium (C). Recombinant TNF-α was used as control. For protein loading control, membranes were reprobed with anti-β-actin antibodies. Data are expressed as the mean of triplicate samples +/− SD of three independent experiments for each patient.

We first confirmed that TNF-α was not released by FLS in response to LPS. FLS (5. 10^5^ cells) were activated with LPS from *Salmonella abortus equi* (1 µg/ml) for 3 h, 6 h and 24 h and activated-cells supernatants were analyzed by ELISA. Although LPS stimulation induced IL-6 secretion (Figure 1A), activated FLS did not release any detectable amount of mature TNF-α in cell culture supernatants (Figure 1B). THP-1 cells were used as a positive control: upon LPS treatment, a high accumulation of TNF-α was found in cell culture supernatants with values reaching 2400 pg/ml (6 h) representing a five-fold increase compared to control (C) (Figure 1C). Finally, we demonstrated by western blotting that non-activated or LPS-activated FLS did not express the mature protein (Figure 1D).

### LPS induced synthesis of TNF-α mRNA in RA FLS

**Figure 2:**
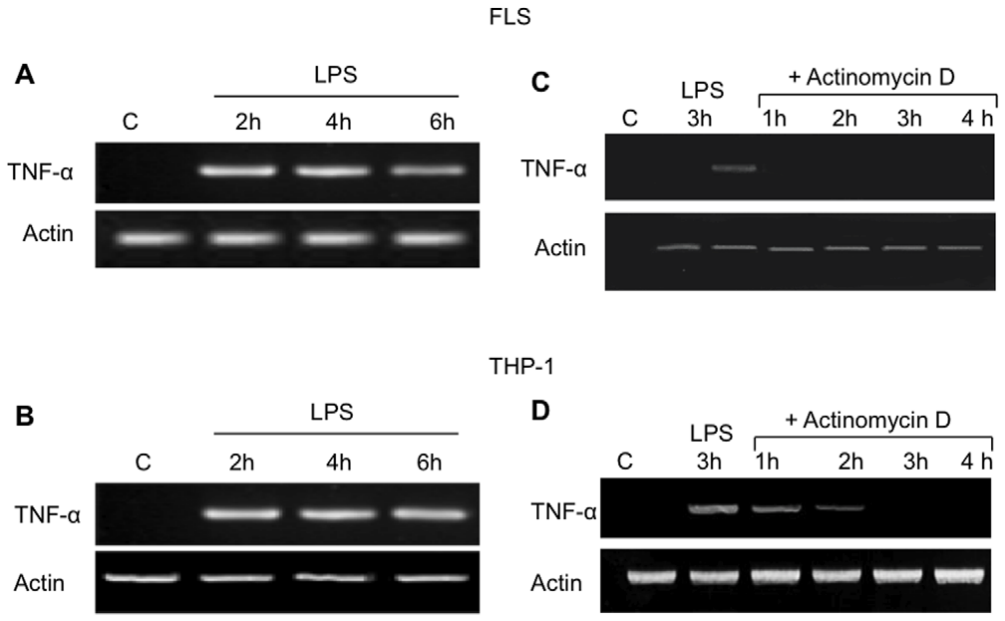
Effect of LPS on TNF-αmRNA expression in RA FLS and THP-1 cells. **A, B.** TNF-α mRNA expression was determined by RT-PCR in RA FLS (**A**) and THP-1 cells (**B**) stimulated with LPS (1 µg/ml) for 2 h, 4 h and 6 h. Control cells were incubated for 4 h with medium (C). **C, D.** RA FLS and THP-1 cells were stimulated for 3 h with LPS and then incubated for another 1, 2, 3 and 4 h with actinomycin D (5 µg/ml). Control cells were incubated for 3 h with medium. TNF-α mRNA expression was determined by RT-PCR. The results are representative of three different experiments for each patient.

We then examined the effect of LPS treatment on TNF-α mRNA accumulation in FLS. RT-PCR was performed with RNA isolated from FLS either non-activated or activated with 1 µg/ml LPS for 2 h, 4 h and 6 h. No constitutive expression of TNF-α mRNA was detectable in control cells, whereas LPS treatment of FLS resulted in a detectable accumulation of TNF-α mRNA within 2 h (Figure 2A). Expression of TNF-α mRNA in FLS was similar to that in THP-1 cells, where TNF-α is normally expressed after LPS treatment (Figure 2B). To investigate the mechanisms responsible for the lack of TNF-α protein synthesis in LPS-activated FLS, cells were incubated with LPS for 3 h and then for another 1 to 4 h with 5 µg/ml actinomycin D. After 1 h of actinomycin D treatment, TNF-α mRNA became undetectable in LPS-activated FLS (Figure 2C) but not in LPS-activated THP-1 cells, where the mRNA accumulation decreased but was still detectable after 1 and 2 h of actinomycin D treatment (Figure 2D). These results indicate that *de novo* synthesized TNF-α mRNA is unstable in LPS-activated FLS.

### TNF-α synthesis is not repressed by miR-125b and miR-939 in LPS-activated RA FLS

**Figure 3:**
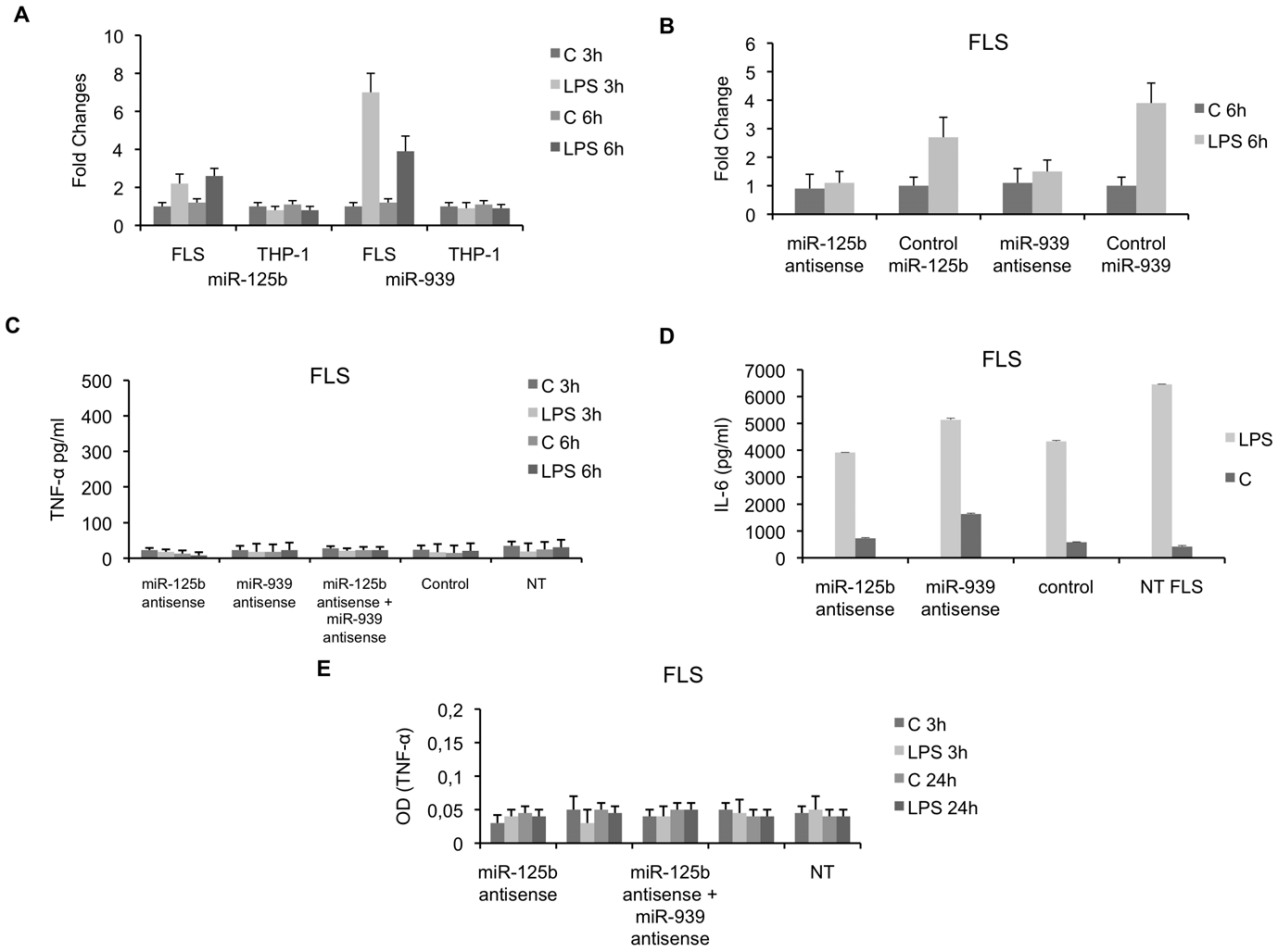
Effect of transfection of miRNA antisense molecules on TNF-αsynthesis by RA FLS. **A, B.** MiR-125b and miR-939 levels were determined by quantitative RT-PCR in RA FLS and THP-1 cells stimulated with LPS for 3 h and 6 h. RNAU6 was used as endogenous control for data normalization. The control (C) corresponded to untreated cells. RA FLS were transfected with either miR-125b or miR-939 antisense molecules or in combination or with the Clear-miR™ negative control (control). LPS or medium (C) activation of transfected cells was performed 24 h post-transfection, for 3 h and 6 h. Non transfected RA FLS were used as negative controls (NT). **C, D.** TNF-α and IL-6 release were determined by ELISA in culture supernatants harvested 6 h after stimulation with LPS or medium (C). **E.** Intracellular TNF-α expression was determined in transfected FLS and activated with LPS for 24 h. Data are expressed as the mean of triplicate samples +/− SD of three independent experiments for each patient.

Using a DNA microarray, we identified two upregulated miRNAs in LPS-treated cells compared to control cells: miR-125b (2.3 fold) and miR-939 (6.1 fold). We confirmed the induction of miR-125b and miR-939 expression by quantitative RT-PCR analysis. The mean increase was 2 and 7 times for miR-125b and miR-939 respectively, after 6 h of LPS-activation (Figure 3A). Similar experiments were performed in THP-1 cells as control, and no changes in the expression of these miRNAs were observed. Since these two miRNAs were predicted to target TNF-α mRNA, we studied the functional consequence of their inhibition by antisense oligonucleotides on TNF-α release by LPS-activated RA FLS. As shown in Figure 3B, transfection of miR-125b and miR-939 antisense molecules impaired endogenous miRNAs expression as compared to miR-125b and mir-939 expression in activated cells transfected with a control antisense oligonucleotide. We next tested whether inhibition of miR-125b and miR-939 affected TNF-α protein release in LPS-activated FLS. We transfected FLS for 24 h with antisense oligonucleotides and then measured TNF-α release by LPS-stimulated FLS. As illustrated in Figure 3C, treatment with LPS did not restore TNF-α release by activated RA FLS transfected with antisense oligonucleotides targeting miR-125b, miR-939 or with a combination of these two inhibitors (Figure 3C). No significant effect of antisense oligonucleotides transfection on cell viability was observed, as determined by MTT assay (data not shown). In addition, the transfection of antisense oligonucleotides did not impair FLS activation since FLS transfected with targeting and non-targeting miRNA inhibitors still produced IL-6 after 6 h of activation with LPS (Figure 3D).

Finally, transfection of antisense oligonucleotides did not restore intracellular or membrane bound TNF-α (Figure 3E). Taken together these data demonstrate that miR-125b and miR-939 are not implicated in the down-regulation of TNF-α synthesis in LPS-activated FLS.

### Inhibition of miR-346 stabilizes TNF-α mRNA but does not induce TNF-α release from LPS-activated RA FLS

We previously showed that miR-346 which is overexpressed in LPS-activated FLS, inhibited Btk expression [25]. As Btk is implicated in TNF-α mRNA stabilization, we therefore hypothesized that the inhibition of Btk expression in RA FLS by miR-346 could result in the subsequent instability of TNF-α mRNA and therefore lack of TNF-α release.

**Figure 4:**
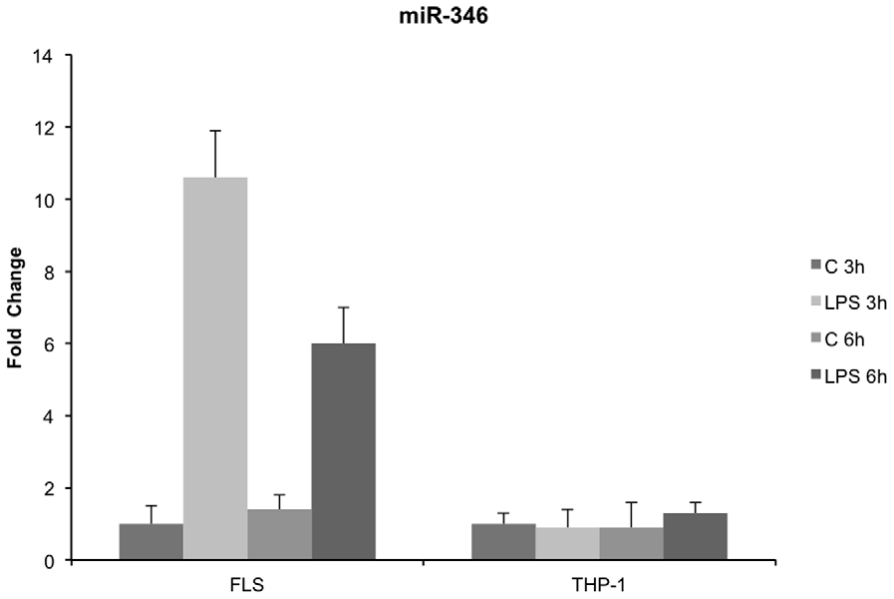
Quantitative RT-PCR analysis of miR-346 expression in LPS-activated RA FLS and THP-1 cells. MiR-346 level was determined by quantitative RT-PCR in RA FLS and THP-1 cells stimulated with LPS (1 µg/ml) for 3 h and 6 h. U6 small nuclear RNA (RNAU6) was used as endogenous control for data normalization. The control (C) corresponded to untreated cells. Data are expressed as the mean of triplicate samples +/− SD of three independent experiments for each patient.

**Figure 5:**
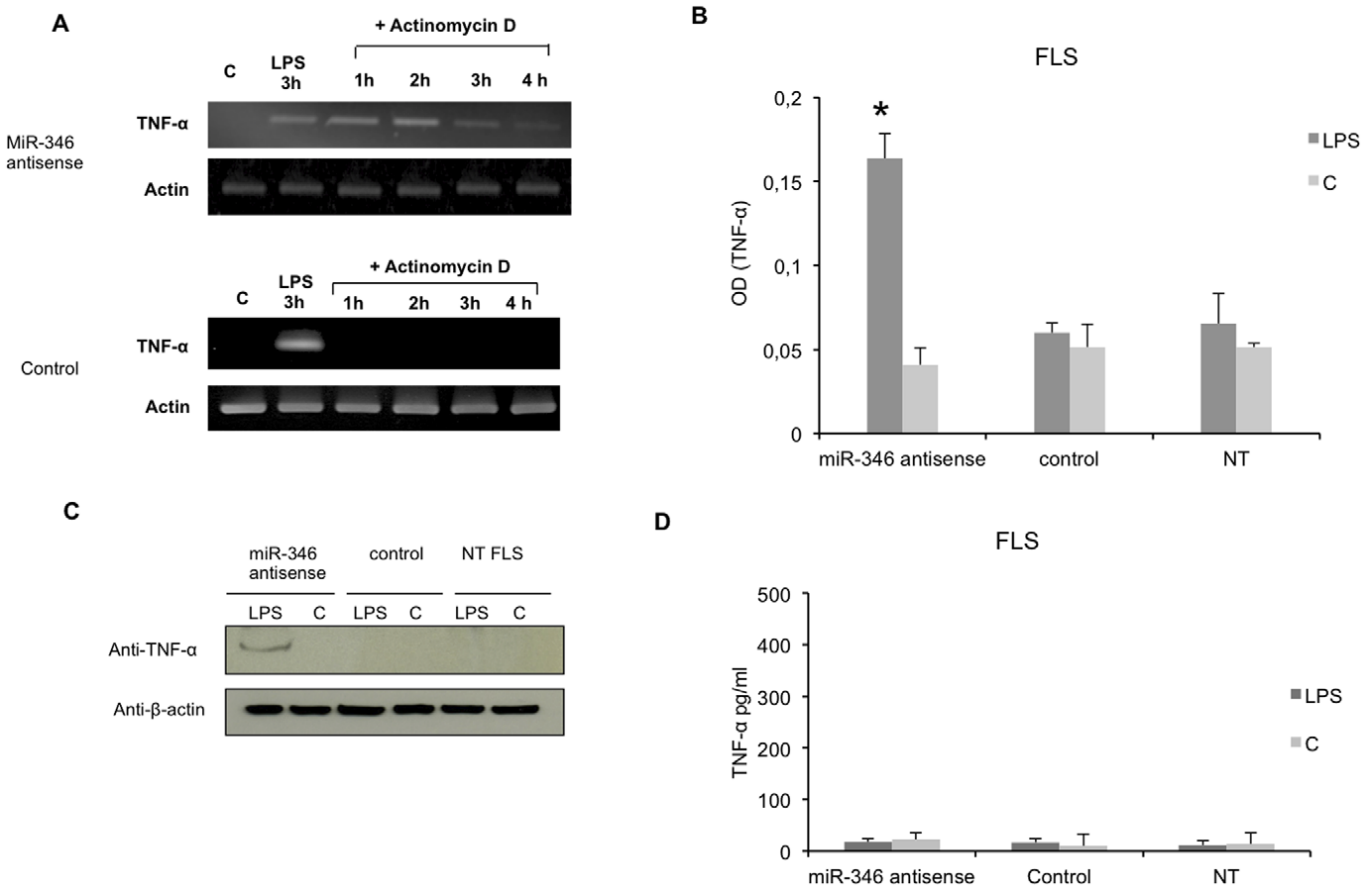
Effect of transfection of miR-346 antagomirs on TNF-α mRNA stability and release in RA FLS. **A.** FLS were transfected with miR-346 antisense molecules or with the Clear-miR™ negative control (control), activated with LPS 24 h post-transfection for 3 h and incubated for another 1, 2, 3 and 4 h with actinomycin D. Control cells were incubated for 3 h with medium (C). NT: non transfected cells. **B, C**: TNF-α expression was detected using cellular ELISA or western blotting with anti-TNF-α antibodies, in FLS transfected with miR-346 antisense molecules or with the Clear-miR™ negative control (control) or in non transfected FLS (NT). 24 h post-transfection, FLS were either incubated in medium (C) or activated with LPS for 6 h. The results are representative of three different experiments for each patient. **D.** TNF-α release was determined by ELISA in culture supernatants after stimulation with LPS or medium (C). Data are expressed as the mean of triplicate samples +/− SD of three independent experiments for each patient. p<0.01.

We first confirmed that miR-346 is overexpressed in FLS but not in THP-1 cells in response to LPS (Figure 4). We then monitored the fate of the TNF-α mRNA in RA FLS transfected with miR-346 antisense oligonucleotides or control antisense oligonucleotides and treated first with LPS for 3 h and then with actinomycin D for another 1, 2, 3 and 4 h. As opposed to the control, we observed a stabilization of TNF-α mRNA after actinomycin D treatment in LPS-activated FLS transfected with miR-346 antisense molecules (Figure 5A). These results indicate that miR-346 plays a role in the control of the stability of TNF-α mRNA. Moreover, we found, using intracellular Elisa, that transfection of FLS with antisense oligonucleotides targeting miR-346, led to the accumulation of intracellular TNF-α after activation with LPS for 6 h. No effect was observed in cells that were not transfected or transfected with a control oligonucleotide (Figure 5B). This result was confirmed by western blotting analysis using anti-TNF-α antibodies (Figure 5C). Nevertheless, treatment with LPS surprisingly did not restore TNF-α release (Figure 5D) or TNF-α membrane expression (data not shown) in activated RA FLS transfected with antisense oligonucleotides targeting miR-346.

### miR-346 and Btk regulate TNF-α release in LPS-activated THP-1 cells

**Figure 6:**
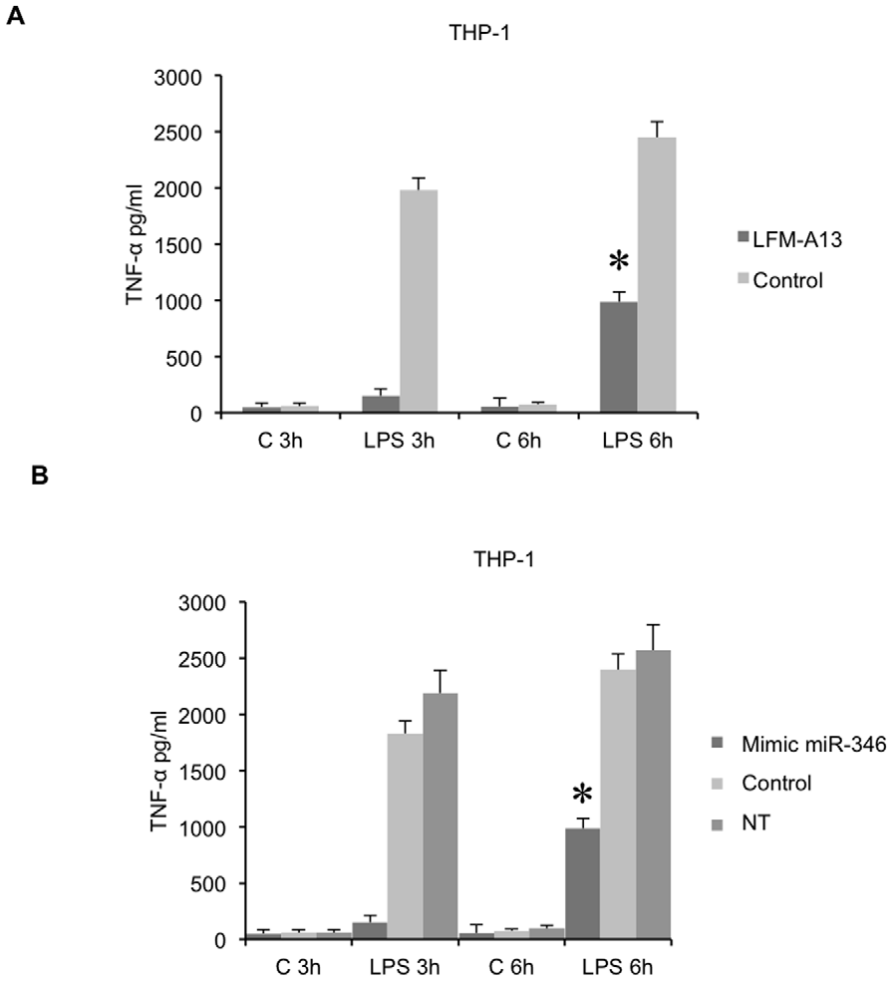
Effect of transfection of miR-346 mimic on TNF-α release by THP-1 cells. **A.** TNF-α release by THP-1 cells preincubated or not (control) with LFMA-13 (172 mM) for 1 h and then stimulated with LPS or medium (C) for 3 and 6 h, was evaluated by ELISA. **B.** THP-1 cells were transfected with miR-346 mimic or with the miRNA mimic negative control (control) and activated 24 h post-transfection with either LPS or medium (C) for 3 and 6 h. TNF-α release was evaluated by ELISA. Data are expressed as the mean of triplicate samples +/− SD of three independent experiments for each patient. p<0.01.

We next tested whether miR-346, which inhibits Btk expression in THP-1 cells [25], could impair TNF-α release from THP-1 cells and act as an anti-inflammatory agent in these cells. We thus examined TNF-α release by THP-1 cells stimulated with LPS and pretreated with Leflunomide metabolite analog (LFM-A13), a known inhibitor of Btk. In these conditions, LPS-induced TNF-α production was strongly inhibited when compared with cells not treated with the inhibitor (Figure 6A). These results demonstrate that TNF-α release is Btk-dependent in THP-1 cells.

We next tested whether the transient expression of miR-346 affected TNF-α release in LPS-activated THP-1 cells. We transfected THP-1 cells with miR-346 mimic and, 24 h later, measured TNF-α release after 3 and 6 h of LPS stimulation. As illustrated in Figure 6B, cells transfected with miR-346 mimic showed a significant decrease of TNF-α release compared to cells transfected with the control miRNA mimic. Altogether, these results confirm that miR-346 participates in the control of TNF-α release in both FLS and THP-1 cells.

### Btk regulates TNF-α synthesis and release in LPS-activated FLS and THP-1 cells by controlling TTP expression

**Figure 7:**
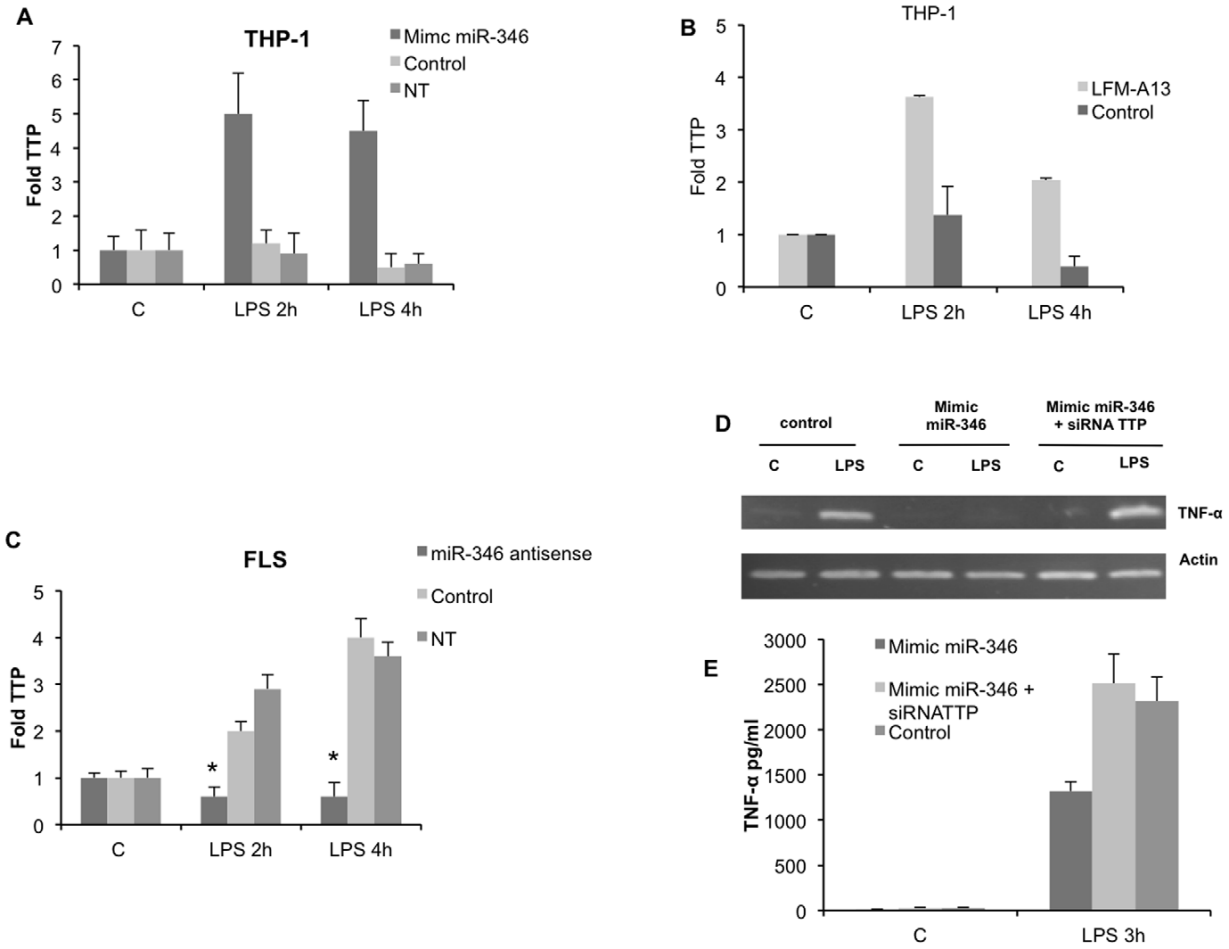
MiR-346 regulates the expression,of TTP in activated RA FLS and THP-1 cells. **A, B:** TTP mRNA levels were determined using quantitative RT-PCR in LPS-activated THP-1 cells transfected with miR-346 antisense molecules or preincubated for 1 h with LFMA-13. **C:** TTP mRNA levels were determined using quantitative RT-PCR in LPS-activated RA-FLS transfected with miR-346 antisense molecules Results were normalized to GAPDH and expressed as fold change compared with samples from cells incubated in medium (C). **D, E:** THP-1 cells were transfected with miR-346 mimic, with miR-346 mimic and siRNATTP or with a negative control (control) and activated 48 h post-transfection with either LPS or medium (C). TNF-α mRNA levels were determined using RT-PCR and TNF-α release was evaluated by ELISA. Data are expressed as the mean of triplicate samples +/− SD of three independent experiments for each patient.

Given the prominent role played by TTP in mRNA decay, we looked into the involvement of Btk on the control of TTP expression. To that end, we transfected THP-1 cells with miR-346 mimic for 24 h and measured TTP mRNA expression after stimulation with LPS for 2 and 4 h. As illustrated in Figure 7A, cells transfected with miR-346 mimic showed a significant increase in TTP expression compared to cells transfected with the control miRNA mimic. We also examined TTP mRNA expression by THP-1 cells pretreated with LFM-A13 before LPS stimulation. In this condition, TTP production by LPS-treated cells was also strongly upregulated compared with non-treated cells (Figure 7B).

We next tested whether inhibition of miR-346 affected TTP expression in LPS-activated RA FLS. We transfected miR-346 specific or control antisense oligonucleotides in RA FLS and treated the cells 48 h later with LPS for 2 and 4 h. We observed a decrease of TTP mRNA expression in the presence of miR-346 antisense oligonucleotides but not with control oligonucleotides (Figure 7C).

To further confirm that miR-346 regulated TNF-α synthesis by controlling TTP expression, we co-transfected THP-1 cells with miR-346 mimic and a siRNA targeting TTP for 48 h and measured TNF-α mRNA expression and cytokine release by transfected cells stimulated with LPS. As illustrated in Figure 7 (D, E), treatment of THP-1 cells with a siRNA targeting TTP stabilized TNF-α mRNA and restored TNF-α release by cells co-transfected with miR-346 mimic as compared to cells transfected with miR-346 mimic alone.

Taken together, these results demonstrate that miR-346 and Btk are implicated in the regulation of LPS-induced TTP expression, which in turn controls TNF-α mRNA stability and TNF-α release.

## Discussion

As opposed to other cells from the synovial membrane, PRRs-activated FLS fail to release TNF-α. This study was aimed at identifying miRNAs that might be involved in the control of TNF-α release. Our results establish that miR-346 can act as a negative regulator of TNF-α release in RA FLS in response to LPS, by inhibiting transcription of the Btk gene. Moreover, since transfection of miR-346 in macrophages also inhibits TNF-α release induced by LPS, these data are a strong indication that miR-346 plays a global role in the control of the inflammatory response.

In an initial attempt to identify miRNAs involved in the control of TNF-α release, we performed a miRNAs microarray analysis of LPS-activated FLS. miR-125b and miR-939 were strongly induced after LPS treatment, and both were predicted to target TNF-α. This finding correlated well with results from Tili et al. [22], who showed that LPS activation of mouse Raw 264.7 macrophages down regulated miR-125b, which allowed TNF-α synthesis by activated macrophages. This result was also in agreement with the fact that overexpression of miR-125b leads to a significant inhibition of the ERK 1/2 pathway without affecting ERK 1/2 levels consistent with the idea that this miRNA acts upstream of ERK [26]. However, inhibition of both miRNAs with antisense nucleotides was not able to restore either TNF-α intracellular expression or release by LPS-activated FLS. Our results suggest that the inhibition of these miRNAs is not sufficient to override the negative regulatory effect induced by LPS signaling on TNF-α release in FLS. Another non-exclusive possibility would be that they may act in FLS in cooperation with other factors.

Previous studies from our laboratory [25], showed that LPS also induces the expression of miR-346 in LPS-activated RA FLS and that this miRNA regulates negatively IL-18 release by inhibiting Btk expression. Btk is involved in the stabilization of various cytokines mRNAs, including the TNF-α mRNA [27]. Moreover TLR2-mediated stimulation of XLA mononuclear cells resulted in impaired production of TNF-α and IL-1β while IL-6, IL-8 and IL-10 remained unaffected through a pathway involving the p38 MAPK [28]. Similar results were observed with dendritic cells from the same patients; stimulation with TLR2, TLR4, TLR7/8 and TLR3 ligands results in lower release of TNF-α IL-6 and IL-12 production was unaffected [29]. On the contrary, in murine bone marrow-derived mast cells, Btk is dispensable in LPS- or lipopeptide-induced secretion of IL-6 and TNF-α [30]. FLS express the epithelial and endothelial tyrosine kinase (Etk), which is implicated in IL-6 and IL-8 release [31], but they fail to express either Btk mRNA or the mature protein constitutively or after LPS activation. Our results established that in FLS, inhibition of miR-346 expression resulted i) in stabilization of TNF-α mRNA and ii) intracellular expression of the mature protein. However this was not sufficient to override completely the inhibition of TNF-α release in cell supernatants. The fact that TNF-α is neither expressed at the cell surface nor secreted indicates that additional mechanisms exist to regulate TNF-α release from activated FLS. There are multiple transport pathways to the cell surface, which depend on the individual cargo. Recently, Lieu et al. showed that secretion of TNF-α in LPS-activated macrophages is controlled by a trans-Golgi network golgin, p230 which is mobilized in response to LPS [32]. It is possible that in RA FLS this mechanism is compromised and additional experiments are now required to resolve this possibility.

Further investigations revealed that LFMA-13, a Btk inhibitor, inhibited TNF-α secretion in THP-1 cells. This result was consistent with other reports, which demonstrated that LFMA-13 inhibited the activation of NF-κB by LPS in THP-1 cells [33]. Moreover, we also demonstrated that transfection of miR-346 mimic in THP-1 cells strongly diminished TNF-α release. As we previously showed that miR-346 inhibits Btk expression in THP-1 cells by an indirect mechanism probably involving yet undefined transcription factors, we propose that in THP-1 cells, the control of TNF-α release by miR-346 involves the inhibition of Btk expression. At present the details of the mechanism that mediate the effect of Btk on TNF-α mRNA stability are unclear. Arsivatham et al. indicated that the components of the ARE-mediated decay pathway are heavily targeted by miRNAs [34]. It appears that ARE control of TNF-α expression may operate at multiple post-transcriptional levels including the miRNAs regulation of ARE-binding proteins.

Among the many ARE-binding proteins that target TNF-α, TTP has emerged as a primary player as it was shown to promote its mRNA lability [11]. As a matter of fact, TTP knocked-out mice show elevated levels of TNF-α production in response to LPS [12]. The question naturally arose whether Btk would be implicated in TTP expression. We showed that transfection of miR-346 antisense oligonucleotides decreased TTP mRNA expression in LPS-activated RA FLS. Furthermore, we also demonstrated that Btk inhibition by either LFM-A13 or miR-346 mimic transfection increased TTP mRNA accumulation in LPS-activated THP-1 cells. These results suggest that stabilization of TNF-α mRNA by Btk is mediated by down-regulation of TTP.

**Figure 8:**
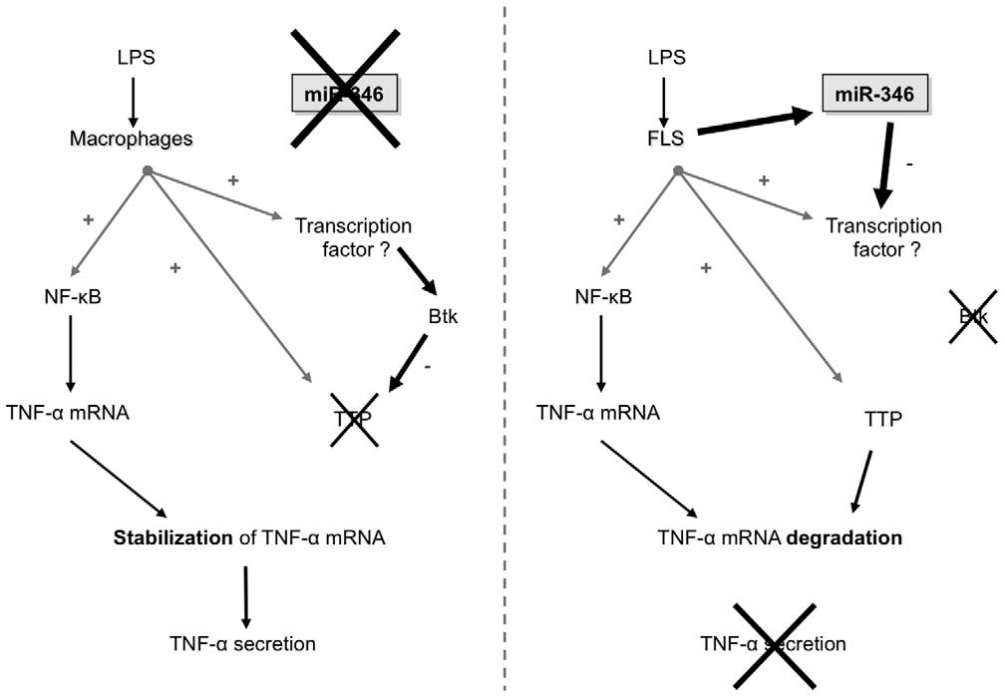
Model of action for miR-346 and Btk in the regulation of TNF-α secretion. See text for details.

We therefore propose a model of regulation of TNF-α release in which LPS induces TNF-α mRNA, miR-346 and TTP expression in FLS. Inhibition of Btk expression by miR-346 would then enable stabilization of TTP expression and would therefore result in the rapid decay of TNF-α mRNA. Blocking Btk either chemically (with the inhibitor LFM-A13), or by overexpressing miR-346 (in THP-1 cells), would therefore result in the inhibition of TNF-α secretion (Figure 8A, B). Taken together, our data indicate an important role for miR-346 in the regulation of TNF-α release by controlling Btk which in turn regulates TTP expression.

In conclusion, our findings provide clear evidence that miR-346 which is induced by bacterial ligands can act as a negative regulator of inflammation in human.

## Materials and Methods

### Reagents

Cell culture media (RPMI 1640, M199 and DMEM), fetal calf serum (FCS), L-glutamine, penicillin, streptomycin, amphotericin B, TRIzol reagent were from Invitrogen (Cergy-Pontoise, France). LPS from *Salmonella abortus equi* and type XI collagenase, actinomycin D, LFMA-13 and anti-β-actin mouse IgG monoclonal antibodies were obtained from Sigma (Saint-Quentin-Fallavier, France). The Lightcycler Faststart DNA Master SYBR Green I was from Roche Applied Science (Penzberg, Germany). The miScript System was obtained from Qiagen (Courtabeuf, France). Clear-miR™miRNA inhibitors were from Eurogentec (Seraing, Belgium). MiRIDIAN® miR-346 mimic and miRIDIAN miRNA mimic negative control and siRNATTP were supplied by Dharmacon (Brebieres, France). Human Dermal Fibroblast Nucleofector™ kit and Cell Line Nucleofector Kit V were from Amaxa (Cologne, Germany). The enzyme immunoassay kits for human TNF-á detection and for human IL-6 detection were from R&D (Lille, France). Anti-Btk mouse IgG monoclonal antibodies were from BD Transduction Laboratories (Le Pont de Claix, France) and anti-TNF-α mouse monoclonal antibodies were from Santa Cruz Biotechnology (Heidelberg, Germany). Throughout this study, buffers were prepared with apyrogenic water obtained from Braun Medical (Boulogne, France). The microarray data were submitted to the Minimum Information About Microarray Experiment (MIAME) database with the accession number E-MEXP-1970.

### Cell culture

Human FLS were isolated from synovial tissues from four different RA patients at the time of knee joint arthroscopic synovectomy as described previously after informed consent was obtained from patients [35]. The diagnosis was conformed to the revised criteria of the American College of Rheumatology [36]. Informed consent was provided according to the Declaration of Helsinki and obtained from all patients. Approval by the ethical committee of the Hopitaux Universitaires de Strasbourg was obtained. FLS cultures were done as previously described [37]. Experiments were performed between the 3^rd^ and the 9^th^ passage. During that time, cultures were constituted of a homogeneous population of fibroblastic cells, negative for CD16 as determined by FACS analysis. Cell number and cell viability were checked by the MTT test (3-(4,5 dimethylthiazol-2-yl)-2,5-diphenyltetrazolium bromide test) as described elsewhere [38]. THP-1 cells (no. 88081201, European collection of cell cultures, Salisbury, UK) were cultured as described previously [39].

### Cell activation

FLS (5.10^5^ cells) and THP-1 cells (10^7^ cells) cells were stimulated with 2 ml of medium alone or medium containing LPS (1 µg/ml) for 3 h, 6 h and 24 h. After stimulation, supernatants were harvested and assayed for cytokine contents using commercially available ELISA tests for human IL-6 and TNF-α.

### Stimulation of cells for total extraction

Total RNA was extracted from human FLS (10^6^ cells) or THP-1 cells (10^7^ cells) incubated for 2 h, 4 h and 6 h with 2 ml of medium alone or medium containing LPS (1 µg ml) using TRIzol according to the manufacturer's instructions.

Total RNA isolated from FLS and THP-1 cells was reverse transcribed using the First-Strand cDNA Synthesis Kit according to the manufacturer's instructions (In Vitrogen) and amplified. For TNF-α, after an initial denaturing at 95°C for 5 min, the temperatures used were 95°C for 30 s, 57°C for 30 s and 72°C for 2 min followed by an extension of 5 min at 72°C. For β-actine, after an initial denaturing at 94°C for 5 min, the temperatures used were 94°C for 1 min, 56°C for 45 s and 72°C for 1 min followed by an extension of 5 min at 72°C. PCR products were separated in 2% agarose gels and visualized with ethidium bromide. The primers used were: (i) TNF-α: 5′- AGC-ACT-GAA-AGC-ATG-ATC-CGG-3′ and 5′- CAT-GGG-CTA-CAG-GCT-TGT-CAC-3′; and (ii) β-actin: 5′-CCA-ACC-GCG-AGA-AGA-TGA-CC-3′and 5′-GAT-CTT-CAT-GAG-GTA-GTC-AGT-3′.

### Real-time quantitative RT-PCR

Quantitative RT-PCR analyses for miRNAs were performed using the miScript System and the primers (Qiagen). RNA concentrations were determined with a NanoDrop instrument (NanoDrop Technologies). Reverse transcriptase reactions and quantitative RT-PCR were performed according to the manufacturer's protocols. A U6 endogenous control was used for normalization. All reactions were run in triplicate in a Lightcycler Instrument (Roche Applied Science). Relative expression was calculated using the comparative threeshold cycle (Ct) method. All reactions were run in triplicate in a using *a Rotor-Gene™ 6000 real-time PCR machine (Corbett Life Science®, Sydney, Australia)*. Relative expression was calculated using the comparative threeshold cycle (Ct) method.

Total RNA isolated from FLS was reverse transcribed using the First Strand cDNA Synthesis Kit according to the manufacturer's instructions (In Vitrogen). Real-time quantitative RT-PCR was performed in a total volume of 20 µl using a SensiMix Plus SYBR® (Quantace, Corbett Life Science®, Sydney, Australia), and gene specific primers:

*GAPDH*: 5′-GGTGAAGGTCGGAGTCAACGGA-3′
and 5′-GAGGGATCTCGCTCGCTCCTGGAAGA-3′
*TTP*: 5′-TCGGGACCCTGGAGCCTGAG-3′.
and 5′ -AGCCAGCGGTGCGAAGCC-3′

TTP and GAPDH were reverse transcribed and amplified. Amplification products were detected as an increased fluorescent signal of SYBR®Green during the amplification cycles. Results were obtained using SDS Software (Perkin Elmer) and evaluated using Excel (Microsoft). Melting-curve analysis was performed to assess the specificity of PCR products.

### Transfections

The Clear-miR anti-miR-125b, anti-miR-939 and anti-miR-346 used in our study were designed to inhibit efficiently the activity of miR-125b, miR-939 and miR-346. They consist of a sequence of 21 nucleotides complementary to the miRNA.

Transient transfection of FLS with Clear-miR™ miRNA inhibitors (200 nM) was performed using the Human Dermal Fibroblast Nucleofector™ kit from Amaxa as previously described [26]. Transient transfection of THP-1 cells with the miR-346 mimic (200 nM) or with miR-346 mimic and siRNATTP was performed using the Cell Line Nucleofector Kit V from Amaxa. FLS and THP-1 cells were then plated in 24-well plates (2.10^5^ cells per well and 10^6^ cells per well respectively). All assays were performed 24 h for miR-346 mimic and 48 h for siRNATTP, post-transfection. Controls were carried out with the Clear-miR™ negative control or with the mimic miRNA negative control. Transfection efficiency was evaluated with the PmaxGFP vector.

Cell numbers and cell viability were assessed using the MTT test. TNF-α release was measured in culture supernatants by a heterologous two-site sandwich ELISA according to the manufacturer's instructions.

### Western Blot

10^6^ cells (FLS) were incubated for various times (3 h, 6 h and 24 h) with LPS (1 µg/ml). Controls were performed with cells maintained in medium with 5% heat inactivated FCS for 6 h. After stimulation, cells were centrifuged and the pellets were suspended 20 min on ice in 300 µL of ice-cold lysis buffer (1% Triton X-100, 20 mM Tris-HCl pH 8.0, 130 mM NaCl, 10% glycerol, 1 mM sodium orthovanadate, 2 mM EDTA, 1 mM phenylmethylsulfonyl fluoride, and protease inhibitors). Lysates were centrifuged for 10 min at 14,000 *g* at 4°C and supernatants were subjected to SDS-PAGE and transferred electrophoretically to nitrocellulose membranes. Membranes were blocked using 1% bovine serum albumin in TBS (20 mM Tris, pH 7.5, 150 mM NaCl) for 1 h at 25°C. The blots were incubated with mouse anti-TNF-α IgG monoclonal antibodies for 2 h at 25°C followed by incubation with horseradish peroxidase-conjugated goat anti-mouse IgG monoclonal antibodies (1 h at 25°C) and detected by enhanced chemiluminescence (Super Signal West Femto Maximum Sensitivity Substrate, Pierce) according to the manufacturer's instructions. To confirm the presence of equal amounts of proteins, bound antibodies were removed from the membrane by incubation in 62.5 mM Tris, pH 6.7, 100 mM β-mercaptoethanol, 2% SDS for 30 min at 50°C and reprobed again with anti β-actin (clone AC-74, Sigma) mouse monoclonal antibodies.

### Detection of cellular TNF-α

Transfected FLS (2.10^4^ cells) with miR-125b, miR-939 and miR-346 antisense oligonucleotides, were seeded into 96-well plates and then incubated 24 h post-transfection in 200 µl of complete medium containing LPS, (1 µg/ml) for 6 h. Cells were fixed with 4% paraformaldehyde in PBS pH 7.4 for 20 min. Free aldehyde groups were quenched with NH_4_Cl 50 mM in PBS pH 7.4 for 20 min. Non specific binding was blocked by incubation in PBS containing either 0,2% bovine serum albumin or 0,2% bovine serum albumin and 0.05% saponin for 30 min at 37°C. The cells were then incubated with biotinylated anti-TNF-α antibodies for 2 h. Absorbance was measured at 450 nm.

### Statistical analysis

Statistical analysis was performed using Student's t-test. Values were compared between different groups in the experiment. P values less than 0.05 were considered statistically significant.
